# Analysis of Structural Changes in the Protein near the Phosphorylation Site

**DOI:** 10.3390/biom13111564

**Published:** 2023-10-24

**Authors:** Kirill S. Nikolsky, Liudmila I. Kulikova, Denis V. Petrovskiy, Vladimir R. Rudnev, Kristina A. Malsagova, Anna L. Kaysheva

**Affiliations:** Institute of Biomedical Chemistry, Biobanking Group, Pogodinskaya, 10, 119121 Moscow, Russia; glucksistemi@gmail.com (K.S.N.); likulikova@mail.ru (L.I.K.); petro2017@mail.ru (D.V.P.); v.r.rudnev@gmail.com (V.R.R.); kaysheva1@gmail.com (A.L.K.)

**Keywords:** phosphorylation, post-translational modification, local change in protein structure, three-dimensional protein structure

## Abstract

Modification of the protein after synthesis (PTM) often affects protein function as supported by numerous studies. However, there is no consensus about the degree of structural protein changes after modification. For phosphorylation of serine, threonine, and tyrosine, which is a common PTM in the biology of living organisms, we consider topical issues related to changes in the geometric parameters of a protein (Rg, RMSD, C_α_ displacement, SASA). The effect of phosphorylation on protein geometry was studied both for the whole protein and at the local level (i.e., in different neighborhoods of the modification site). Heterogeneity in the degree of protein structural changes after phosphorylation was revealed, which allowed for us to isolate a group of proteins having pronounced local structural changes in the neighborhoods of up to 15 amino acid residues from the modification site. This is a comparative study of protein structural changes in neighborhoods of 3–15 amino acid residues from the modified site. Amino acid phosphorylation in proteins with pronounced local changes caused switching from the inactive functional state to the active one.

## 1. Introduction

Over the past two decades, understanding the marker of protein nature in the development of multifactorial diseases has been compromised by a low increase in the number of newly identified markers, despite the technological breakthrough in omics research [1,2]. The high-performance omics technologies have recently spurred the discovery of an incalculable number of candidate biomarkers, but only a negligible fraction of them have become employed in practice [1]. The already discovered biomarkers exhibit a rather low specificity for human health/illness. All these facts limit the development of reliable approaches to early diagnosis.

In recent years, biomedical scientists have focused on aberrant forms of proteins, including modified forms of proteins after synthesis (post-translational modifications (PTMs)) [3,4,5]. It has been noted that approximately 5% of the human genome encodes enzymes responsible for protein post-translational modifications. This fact underlines the importance of PTMs in both normal and pathological conditions [6]. In practical medicine, great hope is placed on modified protein forms [7,8].

Protein PTMs are associated with the development of a wide range of multifactorial diseases from autoimmune diseases and cancer to mental illness [9,10,11,12,13].

Post-translational protein modification is a well-known phenomenon. Over 450 PTM variants have been annotated thus far; most PTMs are catalyzed enzymatically in living systems [14,15], while some occur spontaneously via chemical transformation with involvement of a modifying group (addition, oxidation, or exchange reaction) [8,16]. Omics studies generate a large amount of information, which is a source of data about the association between protein PTMs and pathogenesis. However, they do not provide insights into or understanding of subtle architectural changes that a protein undergoes after modification. That limits the applicability of the information about the presence of PTMs for understanding the pathogenesis at the molecular level. PTMs are covered by several databases (PTMD [17], PhosphoSitePlus [18], dbPTM [19], etc.), which are continually enriched with new data and relevant information about probable associations with diseases. The pool of information accumulated by the scientific community allows for one to study the structural and energetic changes in the protein after modification at the atomic level and predict changes in the biological function of the protein [20,21]. The importance of understanding such protein changes for each PTM variant cannot be overstated; it is used not only in biomedicine but also in the engineering of artificial proteins, including enzymes [22,23].

Many articles focus on identifying the role of changes in the structural organization of proteins as well as the energy states of proteins after modification on their function and their involvement in the pathogenesis of a particular disease [24,25,26,27,28]. It is still an open question whether the changes in protein geometry are significant after modification [29,30]. For instance, Juan Luis Pacheco-García et al. note the loss of stability in human isoform 1 caused by a disruption of the hydrophobic core in the N-terminal domain due to amino acid substitutions or post-translational modification [31]. There are also studies that indicate minor structural changes resulting from protein modifications [30,32,33,34] Fuxiao Xin and Predrag Radivojac showed that the proportion of significant conformational changes (RMSD>2 Å) after glycosylation is estimated to be only 7%, and after phosphorylation, it is approximately 13% [30]. We believe that such a seemingly obvious question can be resolved by consistently and thoroughly studying the effect of each PTM variant on protein structure and function. Using phosphorylation, which is the most common variant of PTM, as an example, we study the aspects of the “life” of a protein without and with modification. Moreover, we try to answer the following question: What changes in protein structure (which probably alter its biological function) are introduced by phosphorylation?

Studies on the structural changes in proteins after modification provide crucial information for predicting the modulation of protein function and determining deeper insights into the significance of PTMs in cellular processes.

## 2. Methods

### 2.1. Study Design

**Figure 1:**
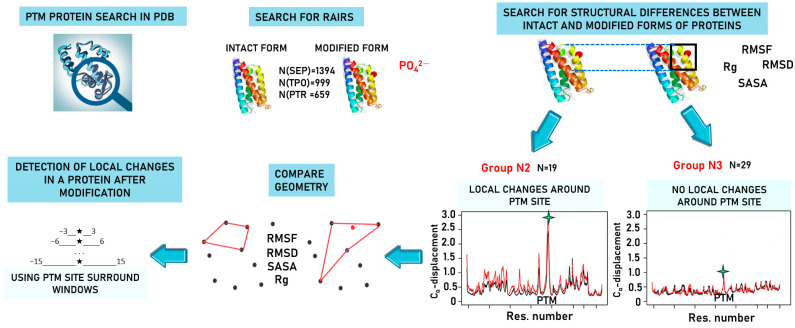
The flow diagram of the study includes the following steps: searching for phosphorylated proteins in PDB, selecting protein pairs (the intact and modified forms), performing comparative analysis of protein geometry for each pair, and detecting local changes in protein structure after modification.

The flow diagram of the study is shown in Figure 1.

Step 1. Selecting phosphorylated forms of proteins according to PDB

The first step of this study was to search for phosphorylated forms of proteins, including those carrying phosphorylated serine (SEP), phosphorylated threonine (TPO), and phosphorylated tyrosine (PTR). The search for proteins that carry at least one modified amino acid residue SEP, TPO, or PTR was performed in PDB (https://rcsb.org, was current as of 22 October 2022).

In this respect, we excluded standard amino acids, nucleobases, common ligands, water, and ions from the search list (standard residues: ‘ALA’, ‘ARG’, ‘ASN’, ‘ASP’, ‘CYS’, ‘GLN’, ‘GLU’, ‘GLY’, ‘HIS’, ‘ILE’, ‘LEU’, ‘LYS’, ‘MET’, ‘PHE’, ‘PRO’, ‘SER’, ‘THR’, ‘TRP’, ‘TYR’, ‘VAL’; nucleobases, common ligands, water, and ions: ‘HOH’, ‘ZN’, ‘CO’, ‘NA’, ‘CU’, ‘MG’, ‘CL’, ‘IOD’, ‘SO4’, ‘HG’, ‘NO2’, ‘UNK’, ‘N’, ‘CA’, ‘C’, ‘G’, ‘A’, ‘U’, ‘I’, ‘DC’, ‘DG’, ‘DA’, ‘DU’, ‘DT’, ‘DI’).

The selection targeted structures with at least one residue unlisted above but that also contain a C-alpha atom (CA). Then, the search was limited to structures with the target non-canonical amino acids SEP, PTR, and TPO (Table 1).

Step 2. Protein pair filtration: the intact and modified forms

At the next step, we solved the problem of PDB filtering of modified protein forms carrying a modifying phosphate group, the respective intact forms carrying no modifying group, and other non-canonical amino acid residues. The dataset should obey the following inclusion criteria:

- The amino acid sequence of the protein must include at least 15 residues (excluding small peptides);
- The protein pair must have a common unique Uniprot ID (excluding comparisons with homologous proteins from different organisms in the intact/modified structure pair);
- The intact form of the pair should not contain any non-canonical amino acid residues (unless the proteins containing non-canonical amino acid variants are matched to the intact protein structure);
- The length of the proteins in a pair should not differ by more than five amino acid residues (excluding comparisons between proteins with significantly different lengths in the intact/modified structure pair);
- The amino acid sequence of the proteins (FASTA) in a pair should match at least 90% (selecting identical sequences in the intact/modified structure pair);
- If several structures are found in the PDB for the modified form, the three-dimensional structure with the lowest RMSD, regarding the intact protein, is selected (reducing the number of comparison variants);
- The number of phosphate groups in the modified form should be ≤3;
- Each protein chain in one crystal with a modifying group is considered a separate structure;
- Structure is identified by a unique combination of UniProt ID and PTM locus (to avoid redundancy in the dataset).

By such filtering, we obtained a set consisting of 63 protein pairs in two forms: the intact and modified ones (Supplementary Materials Table S4).

Step 3. Segmentation of the structures of protein pairs according to their geometric characteristics

The set of selected pairs of protein structures was divided into groups according to the root mean square deviation (RMSD) of C_α_-atoms, which characterizes the changes in geometry after modification of the whole protein (global changes) and modification site (local changes). Two options for analyzing the local changes were considered: (a) different distances from the site, measured in angstroms; and (b) neighborhoods with respect to the modified amino acid residue (±15, ±12, ±9, ±6, ±3). A comparative analysis of the changes in geometric parameters was carried out for each neighborhood of the protein pair modification site.

Table 2 presents the distances calculated for the 63 selected protein pairs (both with and without PTM) for all studied neighborhoods between the modification site and the most remote amino acid in the neighborhood: min, max, median and mean values, and standard deviation (Å). Should neighborhoods of the modification site increase, there is an increase in the absolute distance from the modification site. To simplify the analysis of local changes in protein geometry after modification, we use “windows” of the specific number of amino acid residues from the modification site (neighborhoods of the modification site) instead of absolute distances.

### 2.2. Calculating Geometric Indicators

The input data on three-dimensional protein structures in the “*.pdb” format was prepared to perform subsequent calculations as follows:

- Before calculating the geometric parameters, all the atoms not included in the structure of polymer chains were removed from the structures;
- Hydrogen atoms were removed and then added using PyMol (h_add command) to avoid the error caused by the possible presence of hydrogen atoms in one of the compared structures while being absent in the other one.

Geometric indicators were calculated for the following parameters:

- root-mean square distance between C_α_-atoms in the intact and modified protein forms (RMSD, Å);
- solvent-accessible surface area (SASA, Å^2^);
- radius of gyration (Rg, nm);
- displacement between C_α_-atoms in individual amino acid residues (C_α_ displacement, Å).

The SASA values were calculated in accordance with the Shrake and Rupley algorithm [35] (also known as a “rolling ball” algorithm), implemented in the BioPython library [36], using the default input parameters. The Shrake and Rupley algorithm is a common way amongst bioinformatics tools (BioPython library) to calculate the solvent-accessible surface area of a protein. The approach is based on the drawing of a mesh of points, which are equidistant from each atom of the molecule, and uses the number of these points that are accessible to a solvent to determine the surface area [36].

The RMSD and Rg values were calculated using the tools provided by the PyMol system: the align command (built-in tool to superimpose two structures and find the RMSD value after superimposing) [37] and the gyrate script [38], respectively.

To determine the RMSD and Rg in the neighborhood of the modifying group, we performed segmentation of the amino acid sequence of each protein in the pair in the neighborhoods of ±15, 12, 9, 6, and 3 amino acid residues. The amino acid sequences of the proteins in the pair were re-aligned for each neighborhood. For SASA, the values for the amino acids in the corresponding neighborhood were summarized.

In order to determine the C_α_-atoms displacement of a protein, the structures were also aligned using the align command in PyMOL, and the distances between the corresponding atoms in the structure were calculated.

### 2.3. Analysis of Geometric Indicators

To obtain the general characteristics of the datasets for groups N2 and N3, statistical calculations were performed based on the indicators for the entire structures and for the neighborhood of the modification sites. The Rg and SASA parameters were normalized to the total length of the structure or the length of a specific neighborhood for which they were analyzed (Supplementary Table S4). The percentage of changes in the geometric parameter with respect to the intact protein was calculated using the following formula:

$$∆Rg=\frac{{Rg}_{ptm}-{Rg}_{wt}}{{Rg}_{wt}}*100\%+100\%$$

where Rg_ptm_ and Rg_wt_ are the Rg values of the whole protein or selected neighborhood for the modified and the intact proteins, respectively.

The following formula was used to normalize the SASA parameter:

$$∆SASA=\frac{{SASA}_{ptm}-{SASA}_{wt}}{L_{surr}}$$

where SASA_ptm_ and SASA_wt_ are the SASA values of the whole protein or the selected neighborhood for the modified and the intact proteins, respectively, and L_surr_ is the number of amino acid residues in the neighborhood (the protein length for the whole protein; for a limited neighborhood in proportion to its size: ((±3, 6, 9…) × 2) + 1).

For the RMSD, Rg, and SASA parameters, statistical indicators were calculated, and distribution plots were constructed that contained histograms and kernel density estimation (KDE) curves [39]. The kernel density estimate was calculated using the gaussian_kde function of the scipy.stats package [39]. The bandwidth value for the function was determined automatically for each curve based on the data of 500 points per plot, employing the default Scott algorithm used by SciPy.

## 3. Results

### 3.1. Phosphorylated Proteins in PDB

At the first step, proteins were selected from PDBs containing amino acid residues: phosphorylated serine (SEP), phosphorylated threonine (TPO), or phosphorylated tyrosine (PTR). Filtration revealed 2571 structures carrying at least one non-canonical amino acid from the test set: N_SEP_ = 1394, N_TPO_ = 999, and N_PTR_ = 659. In some proteins, several non-canonical amino acids occur simultaneously, that is, N_SEP_ + N_TPO_ + N_PTR_ > N (Supplementary Materials Tables S1–S3).

**Figure 2:**
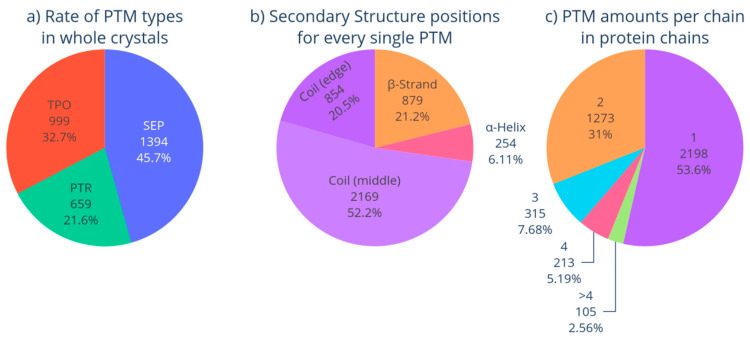
(**a**) The number of proteins in PDB containing non-canonical amino acid residues SEP, TPO, or PTR; (**b**) Variants of the secondary structure of the protein where the modification site is located; (**c**) The number of non-canonical amino acids for each protein chain (see Supplementary Table S3).

Phosphorylation of different amino acid residues (SEP, TPO, PTR) was found to occur unevenly in the dataset (Figure 2a). The modifying groups are contained in all the variants of the protein secondary structure (Figure 2b). The occurrence of non-canonical amino acid residues in proteins is different (Figure 2c).

One can observe in Figure 2a that, for 1394 selected protein structures (being almost 50% of the set of modified proteins), the modification site determines serine (SEP). The non-canonical amino acid TPO was found in 999 protein structures (~33%). The non-canonical amino acid PTR was recognized in 659 proteins (~20%).

Most modification sites are located in unstructured regions (73%); a total of 52% of those are found in the central part of the unstructured region, and 21% are located in the border zone at the junction between the irregular region of the amino acid sequence and the β-strand or α-helix. These non-canonical amino acid residues that are located in the border zone can be misrecognized with STRIDE, DSSP, etc. [40,41]. The occurrence of modification sites in β-strands and α-helices is also quite high: ~21% and 6%, respectively (Figure 2b). Therefore, one can observe that the non-canonical amino acid is located in the unstructured region in slightly more than half of all cases.

The occurrence of non-canonical amino acids in a protein is also heterogeneous. A single modified acid is observed in most proteins (54%); protein variants with two non-canonical amino acids occur less frequently (31%); and those with four or more non-canonical amino acids are even much less common (<8%) (Figure 2c). The largest number of combined modifications was 14 non-canonical amino acids in three proteins (histones). In our study, we considered cases with one, two, and three non-canonical amino acid residues in one chain.

### 3.2. Comparative Analysis of the Geometry of Intact and Modified Protein Forms

The problem of finding a corresponding intact form for each modified protein form was solved in order to study the effect of post-translational phosphorylation on the geometric characteristics of proteins. The corresponding intact pair in PDB could not be identified for every modified protein form. We have obtained a set consisting of 63 pairs (intact and modified forms) of protein structures.

Next, we analyzed the spatial similarity between the whole forms of the proteins in each pair (the so-called “global” changes) as well as the similarity between the neighborhoods of the modification site (“local” changes). The calculated RMSD values characterizing the global and local changes made it possible to segment the set of pairs (n = 63) into three groups (in accordance with the selection criteria listed for step 2 in Section 2.1):

- Group N1 includes protein pairs that are characterized by RMSD > 2Å. Due to significant differences, it is impossible to compare these forms of proteins (see Supplementary Materials Table S2—proteins with RMSD > 2 Å). Fifteen such protein pairs were found. This group of protein pairs was excluded from further consideration;
- Group N2 includes protein pairs of high similarity for which the RMSD value is <2 Å. The protein pairs of this group are characterized by significant spatial differences in the neighborhood of the modification site: the RMSD value of at least one of the studied neighborhoods of the modification site exceeds 2 Å. Nineteen such protein pairs were selected;
- Group N3 also includes protein pairs of high similarity with RMSD <2 Å. The neighborhoods of the modification site are also characterized by high similarity: the calculated RMSD values do not exceed 2 Å for the intact and modified forms. This group included 29 protein pairs.

The thresholds of RMSD less than 2 Å for comparison of structural similarity and more than 2 Å for assessment of structural differences between two or more proteins are commonly used [30,42,43].

Hence, using the performed segmentation, we managed to select protein pairs with local changes or without them after modification, which are of interest to be subsequently studied in terms of their geometric characteristics and for comparative analysis.

**Figure 3:**
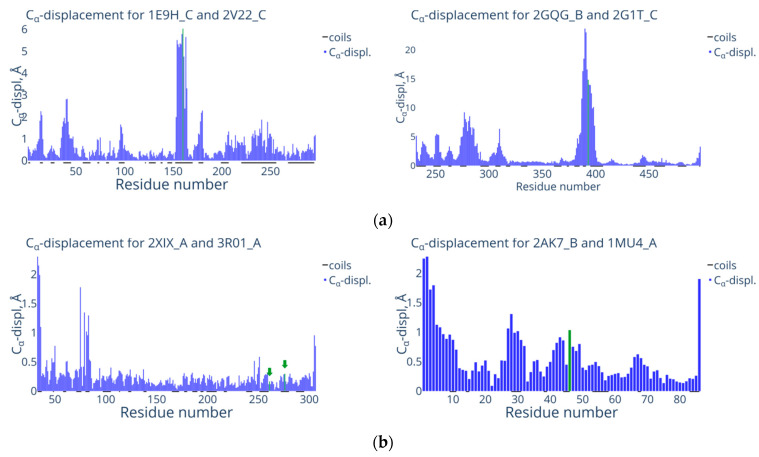
Distribution histograms of C_α_ displacement for the structures from groups (**a**) Group N2 (1E9H chain C—modified form, 2V22 chain C—intact form; 2GQG chain B—modified form, 2G1T chain C—intact form and (**b**) Group N3 (2XIX chain A—modified form, 3R01 chain A—intact form; 2AK7 chain B—modified form, 1MU4 chain A—intact form). The OX axis corresponds to the number of amino acid residues in the sequence; the OY axis corresponds to the C_α_ displacement, Å. Green color and arrows indicate the C_α_ displacement of modified amino acid residues. The regions corresponding to coils (irregular regions of the protein structure) are highlighted in black along the OX axis. Such plots for whole N2 and N3 subsets are available at [44].

Figure 3 shows examples of the typical distribution of C_α_ displacement (distance between C_α_-atoms of every residue in two compared structures) for the protein structures belonging to groups N2 and N3: 1E9H/2V22 and 2GQG/2G1T, 2XIX/3R01 and 2AK7/1MU4, respectively. The histograms for group N2 (Figure 3a) suggest that the modification site is located in the irregular region of the intact form of the 1E9H protein structure and in the short α-helix of protein 2GQG. In both cases, there is a remarkable surge in C_α_-atoms displacements, which is typical for this group near the modification site. This surge is major and exceeds the global changes severalfold (RMSD < 2Å).

A different pattern is observed for the protein pairs belonging to group N3 (2XIX/3R01 and 2AK7/1MU4) (Figure 3b). In the 2XIX/3R01 pair, two modification sites are shown with green arrows: at the junction between the regular region and the constriction and inside the constriction between the regular regions. The absence of a surge in C_α_ displacement for the amino acids near the modification sites is characteristic for the protein pairs belonging to group N3. Hence, for the 2XIX/3R01 and 2AK7/1MU4 pairs, the C_α_ displacement does not exceed 1 Å, i.e., there are no local changes after modification.

**Figure 4:**
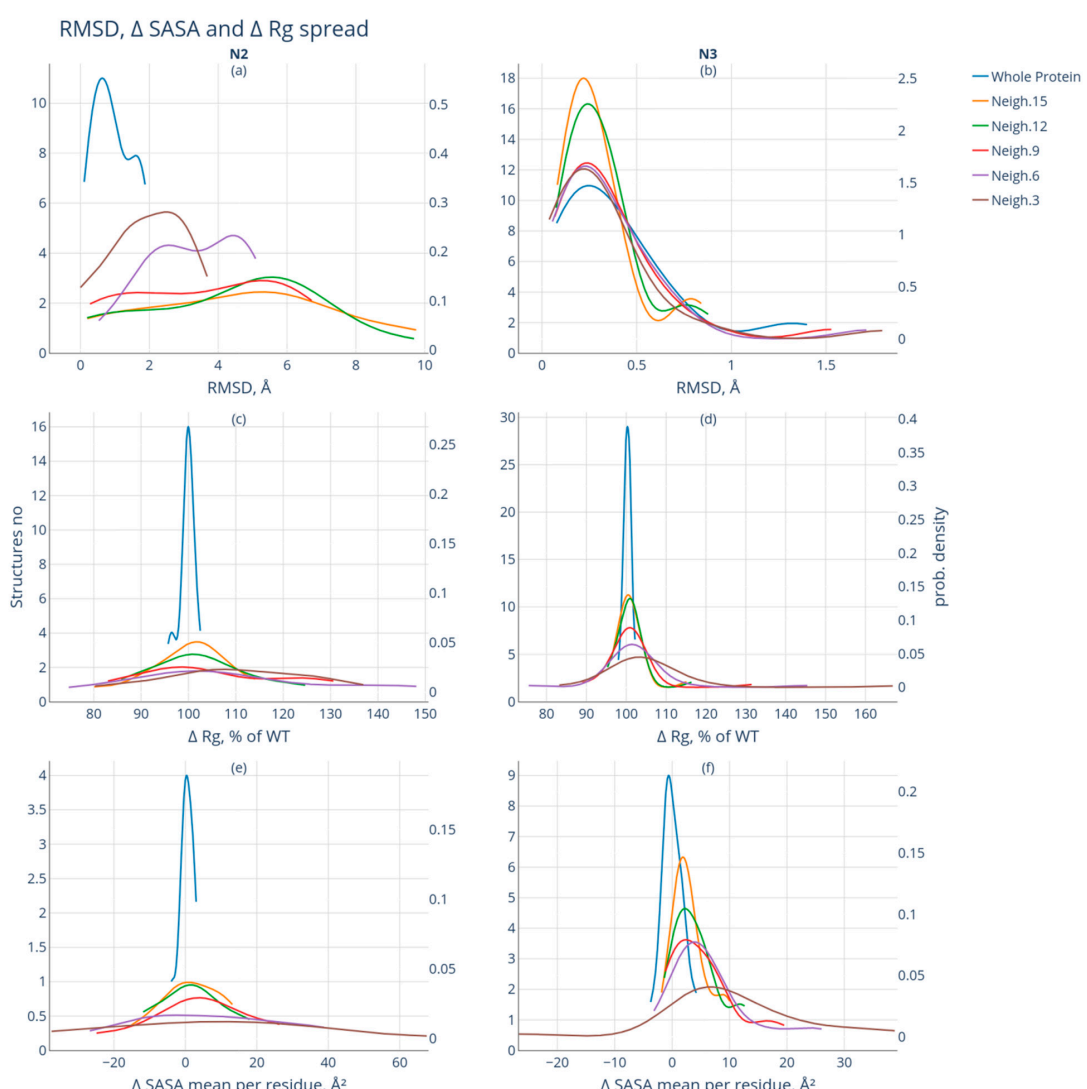
Spread of RMSD, Rg, and SASA values for the neighborhoods of the modification site: ±15, ±12, ±9, ±6, and ±3 (nuclear density estimate). The left column shows the data for group N2; the right one, for group N3. Changes in the studied neighborhood of the modification site of RMSD values (**a**,**b**); Rg (**c**,**d**); and SASA (**e**,**f**). The OX axis corresponds to the RMSD (Å), ∆Rg (%), and ∆SASA (Å2) values, respectively. The Y axis shows the number of structures (left) and the distribution density (right). Correspondence of the curve colors to different neighborhoods is shown in the legend: blue—RMSD, assessment of changes in the geometry of the whole protein after modification; brown—RMSD_3, RMSD for the neighborhood ±3; lilac—RMSD_6, RMSD for the neighborhood of ±6; red—RMSD_9, RMSD for the neighborhood of ±9 a; green—RMSD_12, RMSD for the neighborhood ±12; yellow—RMSD_15, RMSD for the neighborhood ±15.

For both groups of protein pairs, we analyzed the effect of phosphorylation on protein geometry at the local level in the neighborhoods of the modification site: ±15, ±12, ±9, ±6, and ±3 amino acid residues. Figure 4 shows the distribution histograms for the studied characteristics (RMSD, Rg, and SASA) and the smoothing curves for the scatter of the studied parameters (kernel density estimate). Figure 4a indicates that, for the whole proteins belonging to group N2, the distribution curve of the RMSD values has a maximum at ~0.5 Å. The maximum values of RMSD for the examined neighborhoods vary within 3 Å for the ±3 neighborhood, within 4.5 Å for the ±6 neighborhood, and within a range of 5 to 6 Å for the ±9, ±12, and ±15 neighborhoods. Regarding the narrow ±3 neighborhood, the RMSD distribution varies within 0 to 3.5 Å. For the other neighborhoods, a wider range of RMSD values is observed: for the ±6 neighborhood, it ranges from 1 to 5 Å; for the ±9 neighborhood, it ranges from 0.5 to 7 Å; and for the ±12 and ±15 neighborhoods, it reaches 9.5 Å.

Figure 4b shows the distribution of the RMSD values for the whole protein pairs and the studied neighborhoods for group N3 members. One can observe that all the curves lie in the range not exceeding 2 Å with their maxima located at ~0.25 Å. For this group, phosphorylation has virtually no effect on the location of the C_α_-atoms of the amino acids in the immediate neighborhood of the modification site.

The protein’s radius of gyration is its compactness measure: the smaller the Rg value, the more compact the protein structure is. Figure 4c,d illustrate the changes in the Rg values of the whole protein pairs and in the studied neighborhoods of the modification site. In Figure 4c, for group N2, we observe that the distribution curves of the ∆Rg values of the whole proteins have the same maxima at a ∆Rg = 100%; for the ±15 and ±12 neighborhoods, the distribution curves of the ΔRg values have the same maximum at approximately 102%, indicating that the Rg values for the proteins in these neighborhoods increase after modification. It is fair to say that modification in these neighborhoods contributes to a reduction in stable protein packing. There is a wide range of ΔRg values without distinct peaks among the investigated neighborhoods. There is a slight increase (approximately 5%) in the Rg values within the ±3 neighborhood (Figure 4d) in the N3 group. No noticeable changes in the Rg values are observed for the other neighborhoods.

The changes in the mean SASA values of the whole proteins after modification and in the studied neighborhood of the modification site for groups N2 and N3 are shown in Figure 4e and Figure 4f, respectively. The distribution curves of the ΔSASA values for the whole proteins after modification are within the range of (−5; +5) Å^2^ in both groups, indicating a decrease and increase in SASA at once after modification. However, the maximum of ΔSASA for the N2 group is somewhat above 0 Å^2^, while for the N3 group, it is slightly below 0 Å^2^. At the same time, the corresponding curves for the investigated neighborhoods around the modification site in the N2 group have maxima as follows: for the ±15 and ±12 neighborhoods, approximately 0 Å^2^; for the ±9 neighborhood, approximately 5 Å^2^; and no distinct maxima are observed for the ±3 and ±6 neighborhoods (Figure 4e). For the N3 group, an increase in the solvent-accessible surface area is shown for all investigated neighborhoods (Figure 4f). The maximum values of the ΔSASA distribution are approximately 3 Å^2^ for the ±15, ±12, and ±9 neighborhoods; approximately 5 Å^2^ for the ±6 neighborhood; and 8 Å^2^ for the ±3 neighborhood.

**Figure 5:**
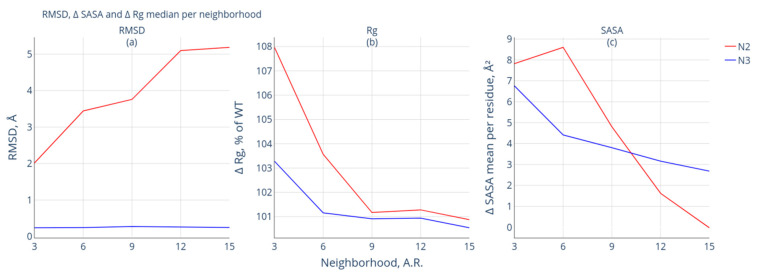
Comparative analysis of the geometric characteristics of the neighborhood of the modification site. The neighborhood of the modification site is plotted along the OX axis: ±15, ±12, ±9, ±6, and ±3. The OS axis corresponds to the values of median RMSD, Å (**a**); median ∆Rg, % (**b**); and ∆SASA, Å^2^ (**c**). The calculated characteristics for groups N2 and N3 are shown in red and blue, respectively.

Figure 5 shows a comparative analysis of the local changes in geometry after modification of the structures (neighborhood of the modification site). One can observe that the response of the neighborhoods of the structures belonging to groups N2 and N3 is different.

The protein pairs belonging to group N3 are characterized by almost the same behavior of structures in all the studied neighborhoods: the median RMSD values correspond to ~ 0.3Å. The response of the structures belonging to group N2 in the neighborhood of the modification site is more pronounced and varies for each neighborhood (Figure 5a). The median of RMSD for the ±12 and ±15 neighborhoods (approximately 5 Å) is higher than those for the other neighborhoods. The minimum value of this parameter is exhibited by the ±3 neighborhood (approximately 2 Å).

Figure 5b illustrates the changes in the Rg values in the studied neighborhood of the modification site. One can observe in the figure that, for the structures belonging to group N2, the median Rg values for the neighborhoods of ±15, ±12, and ±9 (as a percentage) increase by only ≈1% compared to those of the similar neighborhoods of the intact protein. For the ±6 and ±3 neighborhoods, there is a significant increase in the median values of Rg (radius of gyration) after PTM. For the ±6 neighborhood, the increase in Rg is approximately 4%, and for the ±3 neighborhood, it is approximately 8% (the ΔRg value after modification is more than 108%). For the protein pairs in the N3 group, this characteristic for the ±6, ±9, ±12, and ±15 neighborhoods is equal to 101%, indicating a barely noticeable increase in Rg in these neighborhoods. Only for the ±3 neighborhood is the median value of Rg slightly over 103%.

Figure 5c shows the changes in the SASA values in the studied neighborhood of the modification site. For the protein pairs of both the N2 and N3 groups, an increase in SASA is observed for each modification site neighborhood. The maximum increase in the ∆SASA values is characteristic for the ±3 and ±6 neighborhoods: for the N2 group, they amount to approximately 7 Å^2^ and 4.5 Å^2^, respectively, and for the N3 group, approximately 8 Å^2^ and 8.5 Å^2^, respectively. Only a slight increase in the ∆SASA values is observed for the ±12 and ±15 neighborhoods. For the ±12 neighborhood, the ∆SASA values increased by 1.5 Å^2^ for the N2 group and 3 Å^2^ for the N3 group. For the ±15 neighborhood, the ∆SASA values are approximately 0 Å^2^ for the N2 group and less than 3 Å^2^ for the N3 group. Such differences in ∆SASA for the N2 and N3 comparison groups are insignificant, and this parameter does not indicate local changes in the modified protein forms.

## 4. Discussion

Protein PTMs underlie all the signaling pathways, including cell growth and differentiation, transcription and translation processes, aberrant phosphorylation and acetylation, and areas associated with the genesis and development of many human diseases [45].

The most common type of PTM is phosphorylation. Phosphorylated proteins (phosphoproteins) act as regulators of signaling pathways, which makes them attractive targets for the treatments of various diseases, including malignancies, neurological disorders, infectious diseases, and immune disorders [46,47,48,49].

The analysis of structural changes in modified protein forms sheds light on probable changes in protein function and, subsequently, on the mechanism of switching between a healthy cell and the diseased state [10].

There currently is no consensus regarding the effect of covalent modification of a protein on the degree of changes in protein structure geometry and, therefore, changes in its function [30,46].

In our study, we draw attention to the fact that protein phosphorylation can cause structural changes in proteins, which can be found near the modification site. For this purpose, we identified two groups of protein pairs (the intact and modified forms): group N2 (n = 19) that includes pairs of proteins where local structural changes are observed after modification and group N3 (n = 29) for which no local changes after modification were identified.

For both comparison groups, we observed that the modified amino acid can be located both in the regular regions of the amino acid sequence (α-helices and β-strands) and in unstructured regions (coils) (Table 3).

**Figure 6:**
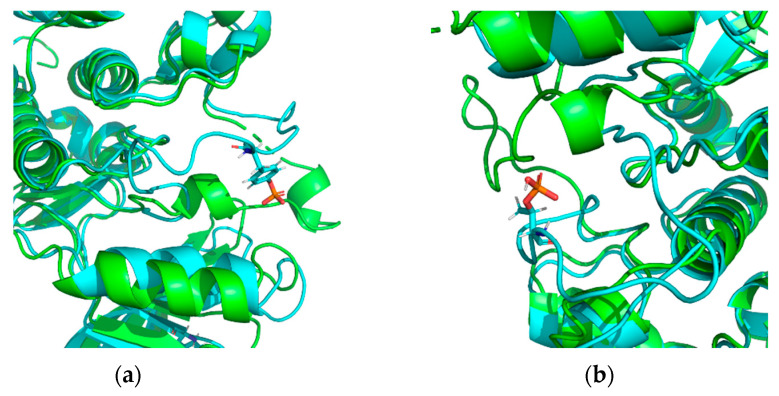
A model of superimposed intact protein (green) and PTM form (light blue) designated on (**a**) proto-oncogene tyrosine-protein kinase Src (PDB ID 1YI6), 416PTR; 416PTR, and (**b**) cyclin-dependent kinase 2 (PDB ID 1E9H), 160TPO.

A modification site located at the junction between regular secondary structure elements and unstructured regions (coils) may provoke a disruption of secondary structure organization near such PTM (the α-helix unwinds, or the β-strand shortens), resulting in the increased length of the unstructured region (Figure 6a). On the other hand, localization of the modifying group in an irregular region (coil) may cause a dramatic change in the spatial position of the coil (Figure 6b).

The analysis of PTM localization displayed that the majority of the PTM incidences of the N2 group occur within unstructured regions, whereas the N3 group is characterized by the incidences predominantly in the α-helix and β-strand, or in the boundary region between the regular secondary structure element and the unstructured region.

The calculations of the changes in the RMSD, C_α_ displacement, and Rg values show this observation for proteins belonging to group N2.

The proteins in the examined groups N2 and N3 have a diverse biological nature. These groups included proteins of different origins, ranging from viral and plant proteins to human proteins (Supplementary Materials, Table S3). The proteins in these groups are enzymes that mostly exhibit the kinase, beta-lactamase, and acyl carrier activities and are involved in a broad range of biological processes: cell cycle, differentiation and division, antibiotic resistance, protein biosynthesis, inflammatory response, as well as lipid and carbohydrate metabolism.

We mapped the binding sites of the partner proteins and other biomolecules for the modified forms of the proteins belonging to groups N2 and N3. Thus, in the considered proteins belonging to group N2, the modification site is located in the immediate neighborhood or within the active site of the protein, or the binding site (see Supplementary Materials, Table S5). For this very group of proteins, there are literature data suggesting that the considered modification types are a natural mechanism for regulating the protein’s biological activity. For example, dual phosphorylation of mitogen-activated protein kinase 13 (PDB ID 4MYG) Thr-180 and Tyr-182 activates the enzyme [47]. Aurora kinase A is also activated by phosphorylation: the protein activation loop carries two adjacent threonine residues, Thr-287 and Thr-288. Phosphorylation of the latter increases the activity of the enzyme [48]. Phosphorylation of Thr-160, as part of the cyclin-dependent activation loop, leads to protein activation [32]. CLK1 activity is positively regulated by autophosphorylation of either tyrosine residues or Ser and Thr residues [49].

Although local structural changes in the group N3 proteins have not been identified, there is information in the literature about the effect of phosphorylation on protein activity. Thus, activation of glycogen synthase kinase-3 beta requires phosphorylation at Tyr-216 in the activation loop [50]. In some cases, the phosphorylation sites of the proteins belonging to group N3 are often remote from the binding sites with partner proteins (see Supplementary Materials Table S5). Therefore, the structural analysis of the changes in the proteins after phosphorylation revealed cases of local changes in the spatial organization of the protein near the modification site (group N2), which possibly alter the biological activity of the considered enzymes. However, it is worth paying attention to some limitations of the statements based on the results of analyzing the protein structures before and after modification. Although we considered absolutely all cases of modified forms of the proteins annotated in the PDB (SEP n = 1394, TPO n = 999, PTR n = 659), proteins with amino acid sequence homology are highly represented in the datasets. This study should be continued as new information about the phosphorylated forms of proteins in PDB becomes available.

## 5. Limitations

Protein Data Bank provides a relatively large collection of three-dimensional structures of phosphorylated proteins. However, this dataset is redundant with individual proteins represented in hundreds of variants, while others are represented only once. Not all phosphorylated forms of proteins have intact forms represented.

The method used for protein pair selection determines the composition of the analyzed dataset. It is important that the structures in a pair can be properly aligned with each other. The selection approach we have chosen is a compromise between sample completeness (attempting to include a maximum of diverse structures) and redundancy avoidance.

There are other criteria and methods that can be used to form and select protein pairs and their combinations, such as the following:

- Filtering without considering differences in structure length with alignment based on the smaller structure;
- Selecting modified and intact protein forms based on sequence homology without considering UniProt ID;
- Disregarding the presence of amino acid substitutions and non-canonical amino acids outside the binding site;
- Considering ligands present in the structures;
- Identifying gaps in the amino acid sequence;
- Selecting protein pairs where both structures were obtained using the same experimental method (X-Ray, NMR, or EM);
- Averaging atomic positions across all three-dimensional structures of a given protein;
- Analyzing local and global structural changes in a protein while considering b-factor values instead of RMSD.

## 6. Conclusions

This work focused on the effect of phosphorylation (the most common type of natural protein modification following synthesis that occurs in all living systems) on protein geometry. The changes in the geometric parameters after phosphorylation, both for the whole protein and in the neighborhood of the modification site, were studied. The work was carried out on a set of protein structures representing all the possible phosphorylated proteins annotated in the PDB. The analysis of the location of modified amino acids in proteins showed that the modified amino acid can be located both in the regular regions of the amino acid sequence (α-helices and β-strands) and in unstructured regions. Furthermore, the modification site can often be located at the junction between unstructured regions and α-helices or β-strands.

We found heterogeneity in the degree (or measure) of protein structural changes after phosphorylation, which allowed for us to isolate a group of proteins with pronounced local structural changes in the neighborhood of up to 15 amino acid residues of the modification site. This study provides a comparative analysis of structural changes (Rg, RMSD, C_α_ displacement, SASA) among the examined neighborhoods of the modification site.

Among all the selected structures, a group of phosphorylated proteins was identified where local structural rearrangements were observed in the immediate environment of the PTM site:

- unweaving or stretching of the α-helix turn, changes in β-strand length, and, therefore, changes in length and conformation of the unstructured region;
- increased Rg values in the studied neighborhood;
- significant surge in Cα displacement values for amino acid residues located near the modification site;
- the calculated RMSD values for the neighborhood of the modification site exceed those for the whole protein.

Therefore, despite the important role of phosphorylation in the regulation of protein activity, structural changes in modified protein forms compared to the intact ones are heterogeneous and vary in their degree.

## Figures and Tables

**Table 1:** Description of the datasets of phosphorylated forms of proteins found in PDB *.

PTM	Number of Protein Structures (PDB)	Mean Resolution, Å	Method	Origin
SEP	1394	2.22 ± 0.8	X-ray: 92% NMR: 3% EM: 5%	Homo sapiens: 53.5% Other eukaryote: 33.2% Bacteria: 6.8% Virus: 0.3% Archaea: 0.3% Other: 1.3% No data: 4.6%
TPO	999	2.21 ± 0.6	X-ray: 93% NMR: 3% EM: 4%	Homo sapiens: 49.1% Other eukaryote: 38.9% Bacteria: 3.9% Virus: 0.5% Archaea: 0.5% Other: 1.7% No data: 5.4%
PTR	659	2.25 ± 0.5	X-ray: 92% NMR: <8% EM: <0.5%	Homo sapiens: 72.9% Other eukaryote: 6.5% Bacteria: 2.4% Virus: 1.7% Other: 0.9% No data: 15.6%

* Complete lists of protein datasets are presented in Supplementary Materials Tables S1–S3 and are available at Nikolsky, Kirill (2023). Result of PDB DB scan for non-standard residues included in protein chains. figshare. dataset. https://doi.org/10.6084/m9.figshare.23564607.v1 (accessed on 23 June 2023).

**Table 2:** Distances between the modified and the boundary amino acid—min, max, median and mean values, standard deviation (Å). Distances have been calculated for the neighborhoods of the modification site: ±15, ±12, ±9, ±6, ±3 amino acid. Measurement unit is Å.

Neighborhood	Min, (Å)	Max, (Å)	Mean, (Å)	Median, (Å)	Std, (Å)
3	5.14	7.61	6.55	6.74	0.57
6	8.77	17.87	13.64	13.86	2.21
9	10.47	26.17	17.8	17.8	3.43
12	11.23	32.86	21	20.14	4.4
15	13.11	37.79	23	20.77	5.78

**Table 3:** Correlation between modified amino acids and elements of secondary structure for the N2 and N3 groups.

Position	Group N2	Group N3
α-helix	1	9
Coil (middle)	13	7
β-strand	3	2
Coil (edge)	2	11

## Data Availability

Kirill Nikolsky (2023). Result of PDB DB scan for non-standard residues included in protein chains. figshare. dataset. https://doi.org/10.6084/m9.figshare.23564607.v1 (accessed on 23 June 2023).

## Supplementary Materials

The following supporting information can be downloaded at: https://www.mdpi.com/article/10.3390/biom13111564/s1, Table S1: “PDB structures with PTM (single PDB entry per line): experimental details and taxonomy”; Table S2: “Protein chains with PTM (single chain per line): amount of PTM residues”; Table S3: “Modified residues (single residue per line): positions and secondary structure assignment”; Table S4: “Protein pairs, wild type and PTM: non-aggregated indicators and values”; Table S5: “Binding sites of partner proteins and other biomolecules for modified forms of N2 and N3 group proteins”.
